# Sensory response following knee joint damage in rabbits

**DOI:** 10.1186/1471-2474-15-139

**Published:** 2014-04-28

**Authors:** Joseph M Hart, Matthew Bessette, Luke Choi, MaCalus V Hogan, David Diduch

**Affiliations:** 1 Department of Kinesiology, University of Virginia, Charlottesville, VA, USA; 2 Department of Orthopaedic Surgery, University of Virginia, BOX 800159, Charlottesville, VA 22904, USA

**Keywords:** Femoral nerve, Afferent, Anterior cruciate ligament, Reconstruction

## Abstract

**Background:**

Altered sensory information arising from damaged knee joint structures has been hypothesized as a contributing factor to persistent muscle dysfunction following injury.

**Methods:**

Composite femoral nerve sensory signal was measured in 24 rabbits randomly allocated (8 per group) to receive surgical anterior cruciate ligament (ACL) transection with or without autograft reconstruction or nothing (control). Two-weeks after the intervention composite afferent signals were recorded from the femoral nerve. Side-to-side ratios (surgical side vs contralateral healthy side) for peak femoral nerve afferent composite signal were used for comparison.

**Results:**

Femoral nerve afferent signal ratios were significantly higher in the ACL-R (2.21 ± 0.74) group when compared to the ACL-T (1.28 ± 0.61, P = 0.02) group and Control group (1.31 ± 0.78, P = 0.03).

**Conclusion:**

The magnitude of sensory information recorded on the femoral nerve is increased following ACL injury and reconstruction surgery, but not after an isolated ACL injury in rabbits.

## Background

Sensory information from mechanoreceptors in peri-articular tissues play a crucial role in proprioception, motor control and dynamic joint stability [1]. Altered sensory information arising from damaged knee joint structures has been hypothesized as a contributing factor to persistent dysfunction following injury such as altered proprioception, force production and coordination [2,3]. In the knee joint, afferent information arises from nerves that innervate muscles crossing that joint. Innervation to the knee joint capsule has been characterized in animal and human anatomic experiments and includes projections from the obturator, saphenous and femoral nerves [4]. Therefore, sensory information from pain, pressure and stretch receptors such as free nerve endings, pacinian corpuscles and ruffini endings would be conveyed along these nerve projections resulting in a composite afferent signal conveyed to the central nervous system. The sensory effects of knee joint injury have been reported in humans indirectly as the underlying cause of arthrogenic muscle inhibition (AMI) [3,5]. In theory, altered sensory information arising from mechanoreceptors within damaged knee joint structures results in an ongoing reflexive response causing a reduction in spinal reflex excitability [6]. Therefore, characterization of a composite afferent signal following knee joint injury or surgery would help develop a better understanding the sensory response to joint injury and possibly explain potential underlying causes of AMI.

Recent investigations have shown the femoral nerve composite sensory signal changes following MCL disruption in rats [7,8]. However, Rabbits have also been used in models of knee injury, muscle weakness and osteoarthritis. The large mammalian nervous system and knee joint anatomy of rabbits provide an adequate model for studying the effects of intra-articular knee ligament injury or reconstructive surgery. Rabbit models of knee injury, osteoarthritis and muscle dysfunction have been described [2,9-14]. For example, quadriceps muscle weakness [11] and atrophy [10] were observed in rabbits with transected anterior cruciate ligaments (ACL) suggesting the role of posttraumatic quadriceps dysfunction in the onset and progression of osteoarthritis. Further, muscle weakness induced by botox injections caused increased cartilage degeneration in rabbits [13]. However, a model to study composite afferent signals arising from the femoral nerve in the presence of ACL injury or reconstruction has not been developed.

Therefore the purpose of this study was to compare femoral nerve afferent signal using a whole nerve recording technique in rabbits 2-weeks following knee joint trauma. We hypothesized that the damage caused by injury and surgery would cause an increase in afferent activity from sensory endings located in tissues within and surrounding the knee joint thereby increasing composite signal measured from the femoral nerve in rabbits with knee joint injury and reconstruction surgery compared to controls due to increase afferent activity from sensory endings.

## Methods

This study was reviewed and approved by the Institutional Animal Care and Use Committee at the University of Virginia. Twenty-four adult, male New Zealand White rabbits were randomized into one of 3 intervention groups. Eight of the rabbits received unilateral ACL transection, 8 of the rabbits received an ACL reconstruction and the remaining 8 were control rabbits who received no intervention. The side receiving the surgical intervention was randomly allocated.

### Surgical intervention

Animals receiving ACL transection (ACL-T) were fully induced with inhaled isoflurane. A knee joint arthrotomy was performed and the anterior cruciate ligament was identified and carefully transected. Care was taken to avoid damage to cartilage or other intra- or peri- articular structures. The joint capsule and skin were closed with absorbable sutures. For the animals in the ACL reconstruction (ACL-R) group the medial third of the patellar tendon was removed and prepared as an ACL autograft. The graft was passed through small diameter tunnels drilled through the tibia and femur and fixed to the tibial and femoral periosteum with suture. Control animals received no surgical intervention and remained on the procedure table for a period of time similar to that of the surgical procedures. The animals receiving unilateral knee surgery (ACL transection with or without reconstruction) were given buprenorphine and a fentanyl patch for pain control and kept in recovery cages on heating pads under warm light post operatively. Animals were housed in a cage for repeat observation on the first day and then daily for signs of pain. Rabbits were all housed for 2 weeks with daily monitoring at which point the terminal measurements were recorded.

After 2 weeks we performed whole nerve recordings on the femoral nerve in each animal bilaterally. This technique was performed in a similar manner to previous published research in rats that utilizing a direct nerve recording technique to characterize changes in composite afferent nerve signal arising from stimulated gustatory receptors [15]. Animals were first prepared by shaving hair around the thighs and groin. Rabbits were fully induced with inhaled isoflurane. We wrapped the lower extremity distal to the knee joint with self-adhesive elastic tape to minimize afferent input from tissues distal to the knee. An anterior incision on the proximal thigh was used to carefully dissect the down to the femoral nerve. The femoral nerve was identified, isolated from surrounding vascular structures and transected to remove efferent signal from nerve recordings. The distal portion of the nerve was de-sheathed using a sharp probe and attached to a platinum recording electrode. A second electrode was placed in nearby muscle tissue to serve as a ground. Signals were passed through a high impedance headstage, amplified and digitized (ADInstruments, Colorado Springs, CO). We recorded neural activity from the femoral nerve during passive knee extension trials. During each passive knee extension trial the knee was extended manually at a constant rate from a flexed position until fully extended.

Afferent signal recorded from the femoral nerve was filtered and integrated and displayed on a computer screen in real time. We continuously monitored visual and auditory signal to assure a consistent baseline level of afferent activity as we manually held the limb to initiate passive movement. Then, the limb was slowly and passively extended to end range and held for 5–10 seconds. The peak signal measured in the extended position was used for analyses.

### Data analysis

Signal was integrated and filtered (50 Hz low pass). The average from 5 trials was calculated then, a ratio between the affected and unaffected side was calculated and used for statistical analysis between surgery groups. The affected side was the numerator and the unaffected side the denominator therefore, ratios higher than 1.0 indicated higher femoral nerve afference on the affected side. For the control group, affected and unaffected sides were selected at random.

### Statistical methods

A 1X3 ANOVA was used to compare femoral nerve afferent ratios among the 3 treatment groups. Tukey’s LSD test was used for post hoc analysis if appropriate. Tests were considered statistically significant if the p-value was 0.05 or less. SPSS version 17.0 (SPSS, Inc., Chicago, IL) was used for all statistical analyses.

## Results

**Figure 1:**
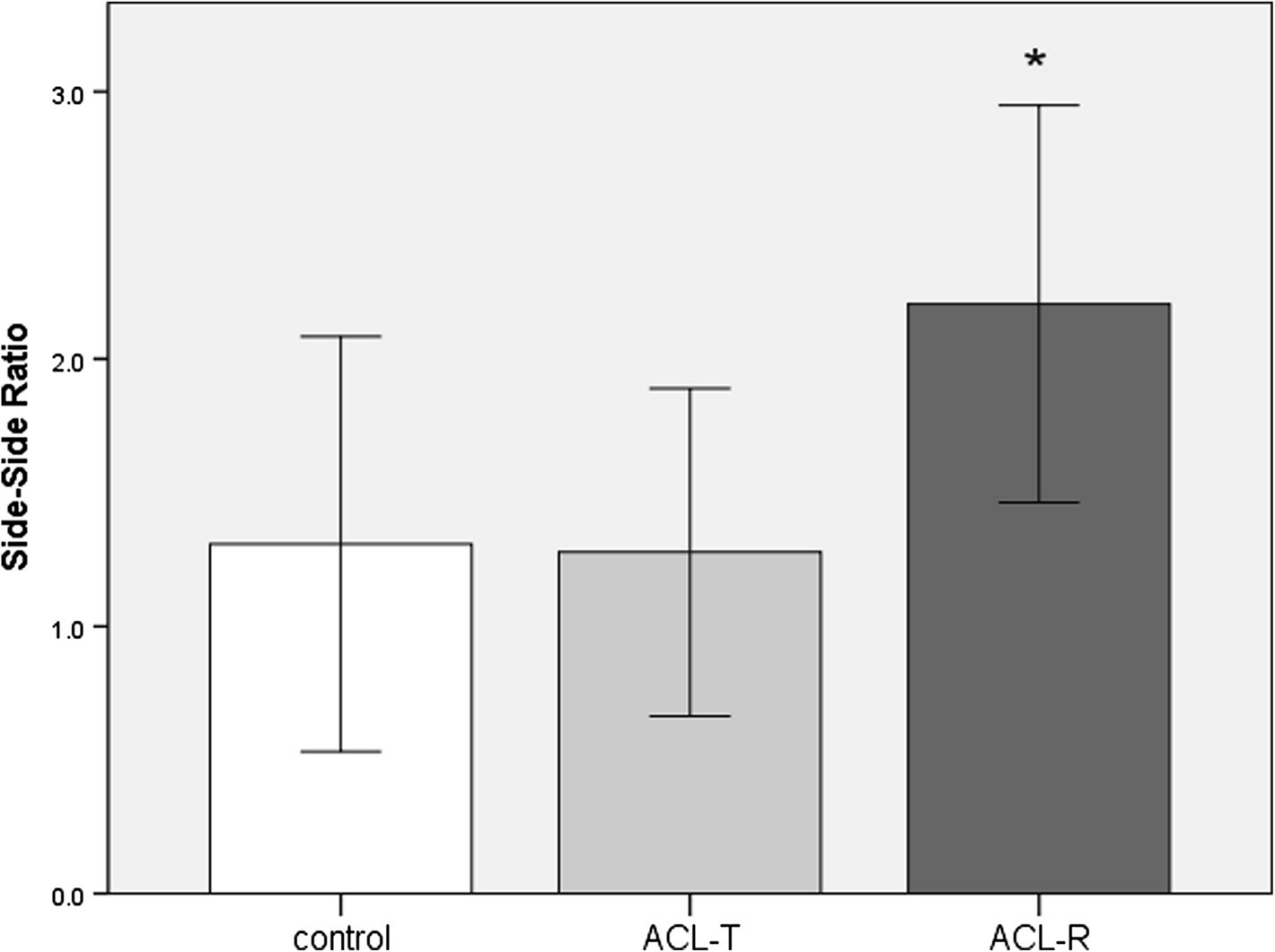
**Average side-side ratios for peak afference measured during passive knee joint extension in rabbits.** Measurements were recorded 2 weeks after ACL transection (ACL-T), ACL Reconstruction (ACL-R) and in animals with healthy knee joints (control). Error bars represent +/- 1 standard deviation. The asterils (*) indicates a significant difference compared to the other groups.

The ratio of peak, integrated femoral nerve afference between the involved and uninvolved sides was significantly different among treatment groups (F_2,19_ = 3.97, P = 0.036). Specifically, the femoral nerve afference ratio was significantly higher in the ACL-R (2.21 ± 0.74) group when compared to the ACL-T (1.28 ± 0.61, P = 0.020) group and Control group (1.31 ± 0.78, P = 0.029). There was no statistically significant difference between the ACL-T group and control group (P = 0.94, Figure 1). Peak nerve recording values are reported bilaterally with side-side ratios in Table 1.

**Table 1:** Peak nerve recordings from the femoral nerve for the affected and unaffected sides in each group*

	**Control**	**ACL-D**	**ACL-R**
Affected side (mV)	14.1 ± 9.9	14.7 ± 6.2	25.5 ± 10.4
Unaffected side (mV)	10.6 ± 4.8	12.0 ± 2.5	11.5 ± 1.9
Side-side ratio	1.3 ± .8	1.3 ± .6	2.2 ± .7

*The affected side was the side that had an ACL transection with or without reconstruction or a randomly selected limb in the control animals.

## Discussion

The primary finding in this study is that composite sensory information measured on the femoral nerve was higher in animals with knee joint injury compared to controls. The differences in side-side ratios were only present in animals in the ACL-R group. The surgical groups were intended to model a scenario of ACL injury with instability versus ACL injury with stability restored through autograft patellar tendon reconstruction. The findings of higher side-side ratios in the ACL-R group suggest that the additional damage created in the knee joint due to the reconstruction procedures resulted in changed sensory information detected in the femoral nerve. Reconstructed knees underwent partial removal of the patellar tendon, potential damage to the joint structures due to tunnel drilling, graft passage and fixation and a more extensive arthrotomy and longer surgical time compared to the ACL transection group. Therefore, these findings suggest that the extent of damage caused by the surgical intervention may explain the observed group differences.

In clinical research, poorer outcomes have been reported in patients with more severe knee joint injuries. For example, higher magnitude quadriceps arthrogenic muscle inhibition has been reported in patients with more severe or extensive joint structure damage [6,16,17]. In addition, patients with recurrent knee injuries such as a failed graft following ACL reconstruction surgery have exhibited poorer self-reported outcomes [18]. Finally, greater impairments in knee joint proprioception was reported in patients with ACL deficient knees reporting instability [19]. Therefore, it is possible that the side-side differences in nerve signal observed in the ACL reconstruction group was due to the addition knee joint damage due to the reconstruction procedures.

In the current study, we observed higher magnitude composite afferent signal in knees that underwent ACL reconstruction compared to control animals. The signal measured from the femoral nerve may include several sources of afferent information. For example, articular structures within and around the knee joint contain nerve receptors such as free nerve endings, pacinian corpuscles and ruffini endings [20,21]. These receptors are innervated by articular branches from the femoral, saphenous, obturator, tibial, common peroneal, and recurrent peroneal nerves [22]. In feline models, it has been reported that the anterior and posterior knee joint capsule is densely innervated with Ruffini endings [23] which are slowly adapting mechanorecptors that respond to capsular stretching [24]. The innervation of the knee joint is dispersed among the various nerve branches. Knee joint innervation has been previously divided into anterior (articular branches from the femoral, common peroneal and saphenous nerve) and posterior (articular branches from the tibial and obturator nerves) [4]. The exact articular distribution is unknown in rabbits, but in other mammalian systems, components of the anterior group of nerve fibers, including the femoral and saphenous nerve articular branches, terminate on structures around the anterior, medial and lateral aspects of the joint capsule and anterior cruciate ligament [4]. Posterior group afferents terminate on posterior structures and the posterior cruciate ligament. Interestingly, branches from the saphenous nerve and the obturator nerve have been reported to form a nerve plexus innervating the posterior capsular structures. In the current study, we certainly did not capture all of the sensory information because we did not record from obturator, tibial or common peroneal nerves. The composite information recorded in the current study likely included information from the femoral nerve and its sensory branch, the saphenous nerve. Therefore femoral nerve signal measured in the current study is most likely attributed to afferent signal arising from articular structures innervated by the femoral and saphenous nerves.

In the current study, we observed higher magnitude composite signal from the femoral nerve in the reconstructed group only. This finding highlights the fact that the anterior cruciate ligament may play an important role in conveying sensory information [4]. Mechanoreceptors such as pacinian corpuscles, golgi tendon organs, and ruffini endings are heavily clustered at the proximal and distal poles of the anterior cruciate ligament [22,25-27] giving rise to afferent proprioceptive information. During an ACL reconstruction, it is likely that terminal branches of sensory nerves are severed as a natural consequence of the surgical procedure so its not clear what sources play a role in conveying sensory information following ACL injury and reconstruction. In the post-amputee literature ectopic afferent, nociceptive signaling has been hypothesized to arise from nerve sprouting from severed nerves [28]. In the current study there may be potential relationship between the loss of tissue and afferent input. For example, reconstruction techniques where the ACL remnants are preserved [29-31] have been described as having good outcomes [32]; attributed to improved improved vascularization and re-innervation due to the ACL remnant [33,34]. The presence of a remnant in the ACL-T group is one differentiating factor that may partially explain why this group did not have increased composite afferent signal. The role of tissue preservation and sensory input is an area for future research.

Joint damage often leads to arthrogenic muscle inhibition in the quadriceps musculature [35]. Arthrogenic muscle inhibition is a unique phenomenon because it exists despite no injury or pathology to the efferent nerve or target muscle. In theory, arthrogenic muscle inhibition is a reflexive response to aberrant sensory information arising from damaged joint structures resulting in a failure to voluntarily activate motor units. The response of Ruffini endings to capsular stretching due to laxity or joint effusion has been implicated in reflexive muscular inhibition that is commonly seen in patients with extensive knee injuries [3,5]. In humans, this manifests as persistent muscle weakness, altered gait patterns and joint degeneration [36]. While this may be a protective response in the acutely injured knee, the long term outcome in persistently inhibited musculature can result in dysfunction. Therefore, if arthrogenic muscle inhibition is persistent following joint injury, recovery may be impeded. In clinical populations, the quadriceps [16] muscle is commonly affected by arthrogenic muscle inhibition which often leads to impaired movement during walking gait [37]. Increased afferent information is currently hypothesized to contribute to post-traumatic muscle inhibition. The findings from the current study may be the basis of future investigations into the potential relationships among increased afferent information and quadriceps muscle dysfunction and osteoarthritis in the post-traumatic knee.

There are some limitations to the study due to the possiblity that other factors associated with knee joint injury, such as inflammation and associated chemical mediators, sensitize afferent nociceptive neurons, which may also contribute to altered sensory information in the post-traumatic knee [38]. However, we feel that the potential influence of chronic inflammation was minimal because upon examining the medical records, none of the rabbits were showing any outward signs of inflammation at the time of follow up evaluations nor any changes in behavior that would indicate the animals were in pain. All were active and healthy at the time of terminal measurements therefore our conclusions are made based on measurements recorded when rabbits were in good health and recovered from their knee joint surgery. Another limitation is the lack of comparison to baseline measurement. Unfortunately, due to the terminal nature of the measurement technique, baseline measurements were not able to be recorded prior to surgery.

## Conclusion

We observed higher side-to-side ratios of peak, composite afferent signal measured directly from the femoral nerve during passive knee extension in rabbits, 2 weeks following ACL reconstruction. This difference suggests higher magnitude sensory information from damaged knee joint structures. This increase in sensory afference may play a role in reflex quadriceps muscle inhibition that is commonly observed in the post traumatic knee.

## Abbreviations

ACL: Anterior cruciate ligament; ACL-R: Anterior cruciate ligament reconstruction group; ACL-T: Anterior cruciate ligament transected group.

## Competing interests

We have no financial disclosures or competing interests relevant to the data presented in this study. The study was supported by internal university funds. All authors declare that they have no competing interests.

## Authors’ contributions

JH designed the study, performed all outcome measures and drafted the manuscript. MB assisted with outcome measures and surgeries, assisted with design and drafting the mnusctript, LC performed the surgeries, interpret data and draft the manuscript, MH assisted with study design, data interpretation and manuscript drafting, DD helped with study design, data interpretation and manuscript drafting. All authors read and approved the final manuscript.

## Pre-publication history

The pre-publication history for this paper can be accessed here:

http://www.biomedcentral.com/1471-2474/15/139/prepub
